# Adaptation of non-technical skills behavioural markers for delivery room simulation

**DOI:** 10.1186/s12884-017-1274-z

**Published:** 2017-03-17

**Authors:** Fabrizio Bracco, Michele Masini, Gabriele De Tonetti, Francesca Brogioni, Arianna Amidani, Sara Monichino, Alessandra Maltoni, Andrea Dato, Claudia Grattarola, Massimo Cordone, Giancarlo Torre, Claudio Launo, Carlo Chiorri, Danilo Celleno

**Affiliations:** 1 Department of Educational Sciences, University of Genoa, Corso A. Podestà, 2, 16128 Genoa, Italy; 2 V.I.E. srl, Spinoff of the University of Genoa, Genoa, Italy; 3 IRCCS Giannina Gaslini Children’s Research Hospital, Genoa, Italy; 4 Advanced Simulation Center, University of Genoa, Genoa, Italy; 5 M.G. Vannini Hospital, Rome, Italy

**Keywords:** Medical Education, Patient Simulation, Education, Obstetric emergency, Social Skills, Clinical Skills

## Abstract

**Background:**

Simulation in healthcare has proved to be a useful method in improving skills and increasing the safety of clinical operations. The debriefing session, after the simulated scenario, is the core of the simulation, since it allows participants to integrate the experience with the theoretical frameworks and the procedural guidelines. There is consistent evidence for the relevance of non-technical skills (NTS) for the safe and efficient accomplishment of operations. However, the observation, assessment and feedback on these skills is particularly complex, because the process needs expert observers and the feedback is often provided in judgmental and ineffective ways. The aim of this study was therefore to develop and test a set of observation and rating forms for the NTS behavioural markers of multi-professional teams involved in delivery room emergency simulations (MINTS-DR, Multi-professional Inventory for Non-Technical Skills in the Delivery Room).

**Methods:**

The MINTS-DR was developed by adapting the existing tools and, when needed, by designing new tools according to the literature. We followed a bottom-up process accompanied by interviews and co-design between practitioners and psychology experts. The forms were specific for anaesthetists, gynaecologists, nurses/midwives, assistants, plus a global team assessment tool. We administered the tools in five editions of a simulation training course that involved 48 practitioners. Ratings on usability and usefulness were collected.

**Results:**

The mean ratings of the usability and usefulness of the tools were not statistically different to or higher than 4 on a 5-point rating scale. In either case no significant differences were found across professional categories.

**Conclusion:**

The MINTS-DR is quick and easy to administer. It is judged to be a useful asset in maximising the learning experience that is provided by the simulation.

**Electronic supplementary material:**

The online version of this article (doi:10.1186/s12884-017-1274-z) contains supplementary material, which is available to authorized users.

## Background

Patient safety during clinical emergencies strongly depends on the effective coordination of multi-professional teams. Such a complex situation cannot be safely managed solely with the implementation of procedures, the adoption of technical skills, and the reliance on high-tech equipment. Recent research on clinical risk management has shown an increasing consensus that the so-called non-technical skills (NTS) are relevant [1–3]. While technical skills are the general procedural and professional competences that are specific to each specialised domain, NTS are defined as the cognitive and social skills necessary to accomplish the technical task in a given situation. They allow practitioners to be aware of the relevant information for the effective treatment of the patient, to make cogent decisions, and to communicate and cooperate with their colleagues in order to achieve high levels of safety and quality of the performance. On the contrary, the lack of NTS has been repeatedly associated with patient injury and medical litigation [4, 5].

Epidemiological data in obstetrics and newborn care support the importance of NTS. According to the World Health Organization, the skilful resuscitation of newborns could save the life of 1 million infants per year [6]. However, the Joint Commission on Accreditation of Healthcare Organizations reports that human factors, communication, situation assessment, and leadership were considered to be the main root causes of the maternal sentinel events that were analysed between 2004 and 2014. Similarly, the same factors were top ranking in the 291 perinatal sentinel events that were analysed in the same time span [7]. Communication breakdown among practitioners or between them and the families has been shown to be associated with perinatal adverse events, patient dissatisfaction, and litigations [8]. Moreover, literature in perinatology reveals that the communication problems mainly concern the unavailability of prenatal information, the difficulty practitioners have in discussing their cases and in sharing doubts with their colleagues, and the inadequate counselling of patients and families [9–11].

In order to address these issues, simulation-based training proves to be an effective strategy to improve the technical and non-technical skills of interdisciplinary teams [12]. There is compelling evidence that simulations can help practitioners to increase team cooperation and communication, critical decision making, and patient care skills [13]. Conventional methods are less effective at teaching and assessing the skills necessary for an interdisciplinary coordination during an emergency, while simulation provides well-designed scenarios that involve the team in complex clinical situations. It can rely on realistic simulators, mimicking the patient as well as the environment. An essential part of the simulation process is the debriefing session on the practitioners’ performance in the simulated scenario. As a training method, simulations have also been extensively used for obstetric emergencies, involving doctors and nurses in teamwork scenarios that concern eclampsia, shoulder dystocia, postpartum haemorrhage, maternal collapse, and cord prolapse [14, 15].

The effectiveness of the simulation method is strongly dependent on the debriefing session. This is because learning in the simulation process occurs when it is based on proper feedback that aims to stimulate both metacognition and reflection on team dynamics. The ability to retrospectively analyse each other’s performances is crucial when it is focused not only on talking about what went well and what did not, but also on why it went well and why something else did not [14, 16]. The key feature of skilled feedback is its capacity to involve the participants in reflecting on the performance without feeling judged or ashamed. Whenever the learners feel the pressure of judgement, their cognitive resources are dramatically devoted to defending themselves instead of integrating the experience into theoretical frames and professional procedures [17, 18]. An adequate method for providing effective feedback during debriefings is focusing on the behaviours observed during the simulated scenario, because any comment will be specific and will refer to actions instead of talking about the person as a whole. The risk of judging personal attitudes is even higher in the case of NTS, where untrained observers could make vague comments on leadership or communication. This feedback would not only distress the learners but it could also be useless, because the recipient would not be able to focus on specific actions that are to be corrected in future circumstances. Therefore, a proper tool for observing specific behaviours during simulation scenarios is of paramount importance, especially for the balanced training of technical and non-technical skills [19, 20]. Even though some tools for the observation of teamwork and communication are present in obstetric literature, there is a lack of structured tools for other professions cooperating in the delivery room (see next section). Therefore, the aim of this study was to develop a tool for the observation of specific behavioural markers for the delivery room teams. Specifically, we designed a NTS inventory to guide the peer observation of behaviour during simulated delivery room scenarios.

## Methods

We first performed an extensive literature review about NTS in healthcare, focusing specifically on the professions involved in delivery room simulation [1, 2, 17, 21–24]. The NTS observation tool for anaesthetists was the only suitable tool for our study already available in literature [25]. We started our investigation on the other professions, taking into account the tools that were developed for similar activities with the purpose of adapting them to the specific professions and the context of delivery room. To investigate the NTS of gynaecologists, we adapted the tool for surgeons [26]. For midwives and nurses, we chose the tool developed for scrub practitioners [27]. We did not find tools for the observation of NTS of healthcare assistants, we therefore developed one anew. Finally, we designed a tool for the overall assessment of obstetrical team performances, since the outcomes are not only due to individual performances but most of all to the interaction among practitioners (see the “NTS Team” form in Additional file 1) [28]. Since we worked with Italian practitioners, we held a series of meetings with representative samples of professionals, thereby working on the adaptation and linguistic translation of the tools to the Italian healthcare context. Each meeting was held by two expert psychology researchers, and involved between two and five practitioners from each professional category (gynaecologist, anaesthetist, midwife/nurse, and healthcare assistant), and followed an iterative design process in order to refine the tool step by step. The main goal was to list only the observable behavioural markers, while avoiding items that were too generic or that were inherent to unobservable mental states [1]. Since the list of items had to be suitable for the Italian context we asked the practitioners to describe their tasks in the delivery room. We tried to develop a tool that was easy to use during the debriefing session of the simulation training aiming to make it quick to complete and easy to understand for practitioners who are unskilled in debriefings and NTS. At the end of the development process, five NTS inventories were available, one for each professional category, which were dubbed MINTS-DR (Multi-professional Inventory for Non-Technical Skills in the Delivery Room).

Each checklist of the MINTS-DR was provided with a short description of the NTS. We defined detailed positive and negative behavioural markers for each skill, in order to anchor the performance rating between two extreme behaviours. In cases where the specific behaviour was not relevant to the scenario, a “not applicable” mark was added to each item. Table 1 shows an example of items for each tool and the tool for global observation of the team is presented as Additional file 1.

**Table 1:** Sample of items for each Non-Technical Skill (NTS) of the MINTS-DR. Only the positive behavioural markers are reported. Some NTS cells are empty because they were not relevant for the specific profession and/or were not reported in literature

NTS	Professional category				Team
	Gynaecologists	Anaesthetists	Midwives/Nurses	Healthcare assistants	
Situation awareness	Requires further resources before they are urgently needed	When clinical conditions change, increases the rate of controls	Activities are controlled and performed according to a priority order	Quickly and properly responds to requests	Quickly reacts to urgent situations
Decision making	The therapeutic options are declared and discussed with the team	Decides the course of actions, comparing the decision with the colleagues	When she makes a decision, declares it and then accomplishes it	-	-
Task management	If the clinical situation changes, reviews the plan of actions	Priorities are defined with the team	Tools and environment are carefully organized	Remains calm even if there is tension in the delivery room	The team adapts to changing situations
Teamwork and cooperation	Begins to operate after checking the availability of tools with the team	Defines the roles among the team before beginning a task	Takes into account and supports other team members’ requests	Provides help and assistance to the others	Roles and responsibilities are clear and rapidly defined
Communication	The plan of action is explained and shared with the team	Talks in a clear, simple and comprehensible way	Requests are direct, motivated and explicit	Listens to the patient’s and family’s requests	Those who get an order confirm the reception
Leadership	When under pressure remains calm and looks for a solution	If an anesthesiological problem occurs, gets control of the situation	-	-	-

All the versions of the MINTS-DR had the same layout. They reported the specific title (e.g., Anaesthetists NTS) and a number from one to three, each representing a different emergency scenario. Each observer was required to create a personal ID code so that his or her answers in the three scenarios were anonymous. Below a brief description on how to mark the items, the form had several boxes, one for each specific NTS. For global team performance, the relevant NTS were situation awareness, task management, teamwork, and communication, as presented in [28]. All the items underwent an iterative refinement process in order to reference only observable behaviours. For instance, instead of asking if every team member knew about his or her role (which is not observable), the item asked if the roles were clearly and explicitly identified.

Each item was made up of a stem sentence, which was as short as possible, followed by two endings. Each ending represented the two extremes of a good or poor performance. For instance, “Communication is… orderly and directed to specific team members/not specifically directed and chaotic (many people talking at the same time)”. The good performance behavioural marker was on the left and the poor performance behavioural marker was to the right of the scale. On the rightmost side, the NA option was present, in case that specific item was not applicable to the situation. The two behavioural marker extremes were presented as anchors on a 4-point Likert scale. We adopted this kind of scale because, in a previous test, we observed that a dichotomous scale was perceived to be too judgemental by respondents, since they only had two options, i.e., good or poor performance. Therefore, this layout had the potential to bias the observations towards a high proportion of “good performance” ratings. A three-point scale, on the other hand, will provide a mid point, in between “good” and “poor”, but we decided to discard this option because of the risk of getting too many uninformative middle ratings. As we said before, the risk of being judgmental is high when providing feedback on their colleagues’ performances. We wanted the observers to feel comfortable in providing feedback that was both easy to express and useful to understand the actual observed behaviour. Therefore, we adopted a four-point scale, which presented two extreme behaviours and two intermediate levels, which were milder but still informative enough to help the facilitators to trigger an open discussion during the debriefing.

The usefulness and the usability of the MINTS-DR were then tested. A total of 86 health care workers served as evaluators. The sample was made up of 18 anaesthetists, 12 gynaecologists, 39 nurses/midwives, and 17 assistants (females 80%, mean age 40.74 ± 10.41 years, range 24–61), who worked at the IRCCS Giannina Gaslini of Genoa, one of the main paediatric hospitals in Italy. They were involved in an 8-h training course that was based on the simulation of obstetric haemorrhage. The course took place at the Advanced Simulation Centre of the Medical and Pharmaceutical Sciences School of Genoa, University of Genoa, and it was based on the high fidelity NOELLE® S574.100 Tetherless Maternal and Neonatal Birthing Simulator. We performed a total of 5 courses, each containing between 7 and 10 participants, while ensuring that each profession was represented.

The course started with an introduction to crisis resource management, non-technical skills and the role of simulation in emergency and risk management. During this introductory phase, the participants performed a training session to get familiar with the use of the NTS checklists. The simulation training was based on three scenarios that were developed in order to pinpoint specific emergency situations and trigger team dynamics based on the proper adoption of NTS. The scenarios were:

1. Post-partum haemorrhage due to uterine atony;
2. Eclamptic seizure and haemorrhage in pregnant woman affected by severe preeclampsia;
3. Uterotonic drug management during peripartum haemorrhage.

Each scenario lasted 15–20 min. All the participants were involved in at least one scenario, while their colleagues were observing the performance on a display screen in a separate, quiet room. The screen displayed the scene from three points of view and reported the clinical parameters of the woman and the foetus (heartbeat, oxygen peripheral saturation, non-invasive blood pressure). A team of simulation experts, representative of each professional category, remotely controlled the clinical parameters and the woman’s voice. In some scenarios, a faculty member played the role of the father attending the delivery. Before the scenario began, the observers were given the NTS checklists according to their profession and were instructed to focus their attention on their colleague’s performance. One of the observers, instead of observing his or her colleagues, was asked to use the team observation checklist. After each scenario, the observers filled in the respective checklists and then were asked to provide feedback to their colleagues during the debriefing, referring to their forms. At the end of the course, all the participants rated the usability and usefulness of the MINTS-DR on a 5-point rating scale; scale (1 = “not at all”; 2 = “slightly”; 3 = “average”; 4 = “moderately”; 5 = “completely”). We considered mean ratings of no less than 4 on either characteristic as a satisfactory result [29].

In order to test whether the usability and the usefulness ratings of the MINTS-DR reached the abovementioned level in each professional category, we used one-sample *t*-tests, setting the test value to 4. We then tested for differences across professional categories using Linear Mixed Models (LMMs). LMMs were preferred over a one-way ANOVA since we needed to take into account the nesting of participants into courses.

## Results

We administered 86 MINTS-DR for the observation of NTS and comments on them during the debriefing. Descriptive statistics of the ratings of usefulness and usability are reported in Table 2.

**Table 2:** Descriptive statistics of the usefulness and usability ratings of the MINTS-DR

Variable	Total sample (*n* = 86)	Anaesthetists (*n* = 18)	Gynaecologists (*n* = 12)	Midwives/Nurses (*n* = 39)	Healthcare Assistants (*n* = 17)	Test of differences across groups
Usefulness	4.34 ± 0.65 (3–5)	4.33 ± 0.69 (3–5)	4.09 ± 0.70 (3–5)	4.33 ± 0.62 (3–5)	4.53 ± 0.62 (3–5)	F(3, 81.95) = 1.06, *p* = .369, η^2^ = .01
t	4.82	1.97	0.43	3.28	3.42	
df	85	17	11	38	16	
p	.000	.065	.678	.002	.004	
d	0.52	0.46	0.12	0.53	0.83	
Usability	3.90 ± 0.85 (2–5)	3.89 ± 0.90 (2–5)	3.75 ± 0.87 (2–5)	3.77 ± 0.87 (2–5)	4.29 ± 0.69 (3–5)	F(3, 86) = 1.75, *p* = .162, η^2^ = .02
t	−1.08	−0.50	−0.95	−1.63	1.68	
df	85	17	11	38	16	
p	.281	.621	.361	.111	.112	
d	−0.12	−0.12	−0.28	−0.26	0.41	

Notes: Values are mean ± standard deviation; bracketed values represent the range of scores; t: t-value from the one-sample *t*-test (test value: 4); df: degrees of freedom from the one-sample *t*-test; p: p-value from the one-sample *t*-test; d: Cohen’s d from the one-sample *t*-test

Table 2 shows that none of the ratings fell below 2 (“slightly”). Taking into account the rating of usefulness in the total sample, one-sample *t*-tests showed that the ratings of midwives/nurses and healthcare assistants were statistically higher than 4, while anaesthetist and gynaecologist ratings were not statistically different to 4 (Table 2, upper panel).

Concerning usability, one-sample *t*-tests revealed that all the ratings were not statistically different to 4 (Table 2, lower panel).

Results revealed that ratings did not statistically differ across the professional categories (Table 2, rightmost column).

Taken together, these results suggest that groups of practitioners positively (mean ratings never fell under 4) and uniformly (no differences in mean ratings across groups) rated the usefulness of MINTS-DR during the debriefing and its usability as a tool for NTS observation during the simulation.

## Discussion

The results suggest that the MINTS-DR behavioural markers are a coherent support for the debriefing among peers, since they help them to observe and discuss NTS by grounding their feedback in objective behaviours. This method proves to be one of the most promising ways to enhance metacognition and to learn about complex and ambiguous topics such as NTS. Among the limitations of this study, we mention the lack of an objective pre- and post-assessment of the debriefing quality. However, we observed that prior to the introduction of MINTS-DR the debriefing was often unbalanced. This is due to the discussion about non-technical and relational aspects being rather vague and, most of all, not providing clear positive behavioural benchmarks after a detailed reflection on technical aspects (e.g., the medical guidelines for the haemorrhage treatment). This part of the debriefing was generally conducted within the frame of reference of the Crisis Resource Management (CRM) checklist, provided by Gaba and colleagues [30]. The checklist is a comprehensive inventory of NTS such as “Communicate effectively”, “Allocate attention wisely”, “Know the environment”, etc. These key points are coherent with literature research on NTS. However, they are purposefully broad so as to tackle many situations and several professions involved in healthcare crisis management. During the debriefing these key points were listed but a reference to specific behavioural markers for the delivery room activities was lacking. Sometimes, observers had trouble linking each general item of the CRM list to the specific performance and, on the other hand, the participants in the simulation were not helped to understand to what specific behaviour an “effective communication” referred to. Facilitators had to focus on this gap between behaviours and general principles, helping participants in the metacognition process. Unfortunately, observers were seldom engaged in this process and provided only a few generic comments on their colleagues’ performance. The introduction of MINTS-DR, instead, moved the locus of control from the facilitators to the observers. The facilitators still had the responsibility of the quality of the debriefing, but their role was more subtle and just supported the observers in providing their feedback through the MINTS-DR and triggering the participants’ metacognition when necessary. This way, the MINTS-DR was not just a tool for the debriefing, but became a frame of reference for all the clinical staff who took part in the simulation. The behavioural markers were not simply used for the feedback, but were internalized by participants and observers. In addition, enabling the colleagues to provide specific feedback for each other would extend the effectiveness of the simulation beyond the time frame of the scenario and the debriefing, becoming common ground for open discussion in the workplace.

## Conclusions

The present research aimed to introduce a coherent and comprehensive tool for the observation of behavioural markers of NTS in the delivery room simulation. Simulation in healthcare is becoming the cutting edge of practitioner training and education. However, it is important to capitalize on this method by means of a proper debriefing session, where the experience could be integrated with the reflection on theories, models, guidelines, and expected behaviours. Given the paramount importance of NTS for patient safety, a sound reflection on these skills is needed during the debriefing. In order to address this need for delivery room practitioners, we developed the MINTS-DR, a quick and easy tool for the observation and assessment of NTS (average completion time: 5–10 min) for the multi-professional cooperation. We developed an observation form for anaesthetists, gynaecologists, nurses/midwives, assistants, and a global team assessment. Each form was designed according to a bottom-up process, starting from interviews with practitioners and adapting the items to the national operative context. The results showed that these tools were well accepted by all the professional categories involved in the delivery room. However, more research is needed in order to evaluate the psychometric properties (i.e., inter-rater reliability, sensitivity, and coherence) of the MINTS-DR, its learning effectiveness, and its ability to actually improve delivery room NTS.

## Additional file

Additional file 1: NTS Team Behavioural markers observation form for the team performance in the delivery room. (PDF 170 kb)

## Abbreviations

MINTS-DR: Multi-professional Inventory for Non-Technical Skills in the Delivery Room
NTS: Non-technical skills
