# Supplementary material for: Adaptation of non-technical skills behavioural markers for delivery room simulation
Source: BMC Pregnancy Childbirth. 2017 Mar 17;17:89. doi: 10.1186/s12884-017-1274-z (PMC5356378; doi:10.1186/s12884-017-1274-z)
Supplement: Additional file 1: — NTS Team Behavioural markers observation form for the team performance in the delivery room. (PDF 170 kb) [file 12884_2017_1274_MOESM1_ESM.pdf]

University of Genoa
School of Social Sciences
Department of Education Sciences

DISFOR

NTS TEAM

SCENARIO N.______

ID code | | | | | |

For each item, please choose the option that in your opinion most accurately describes what you observed during the simulation. If the item is not applicable to the observed scenario (for instance, it was not necessary to call for help), please mark the NA option.

SITUATION AWARENESS

Attention…	is focused on the task	O O O O	is directed away from the task	NA
The team is in control of…	the whole situation	O O O O	just a single event	NA
The request for help is…	timely	O O O O	delayed or absent	NA
The acknowledgement of one's own limits (knowledge, skills, competence) is…	timely and followed by precautionary actions	O O O O	slow or absent	NA
The response to urgent situations is…	timely	O O O O	slow or absent	NA

TASK MANAGEMENT

Priorities are…	explicitly identified	O O O O	not explicitly declared	NA
Goals are…	set and communicated	O O O O	not clearly set or shared	NA
Team members act…	assessing the availability of resources	O O O O	without planning	NA
When the situation changes, the team…	adapts its behaviour	O O O O	is uncertain and hesitant	NA

©Fabrizio Bracco 2017 – DISFOR, University of Genoa

**TEAMWORK**				
The leader...	is clearly identified and acknowledged	O O O O	is not clearly identified	NA
Roles and responsibilities are...	clear and rapidly identified	O O O O	ambiguous and/or slowly identified	NA
The destructive behaviour of team members (e.g., shouting, being rude, etc.) are...	actively managed	O O O O	ignored	NA
The atmosphere in the room is...	controlled	O O O O	chaotic (raucous, lack of listening, unmanaged alarms)	NA
Team members...	help each other and share ideas	O O O O	operate as independent and uncoordinated	NA

**COMMUNICATION**				
Communication is...	orderly and directed to specific team members	O O O O	not specifically directed and chaotic (many people talking at the same time)	NA
Whoever gets an order...	recognizes it	O O O O	does not explicitly recognize it	NA
Whoever completes an order...	confirms having completed it	O O O O	does not confirm its completion	NA
The requests for help are...	taken into account	O O O O	ignored	NA
How many team members introduce themselves to the patient?	all of them	O O O O	nobody or only some of them	NA
The patient/partner...	are informed and involved in the decisions	O O O O	have to ask what is going on	NA
Patient's anxiety/hostility is...	well managed	O O O O	neglected	NA

NA = Not Applicable

©Fabrizio Bracco 2017 – DISFOR, University of Genoa
